# Preferences of oral nutritional supplement therapy among postoperative patients with gastric cancer: Attributes development for a discrete choice experiment

**DOI:** 10.1371/journal.pone.0275209

**Published:** 2022-09-29

**Authors:** Qiuchen Wang, Yahong Chen, Yi Peng, Hua Yuan, Zhiming Chen, Jia Wang, Hui Xue, Xiuying Zhang

**Affiliations:** 1 Department of Fundamental Nursing, School of Nursing, Jilin University, Changchun, Jilin Province, PR China; 2 Interventional Operating Room, China-Japan Union Hospital of Jilin University, Changchun, Jilin Province, PR China; 3 Department of Hematology, First Hospital of Jilin University, Changchun, Jilin Province, PR China; 4 Department of Histology and Embryology, College of Basic Medical Sciences, Jilin University, Changchun, Jilin Province, PR China; University of Huddersfield, UNITED KINGDOM

## Abstract

**Background:**

Adherence to oral nutritional supplement therapy among postoperative patients with gastric cancer is low. There is little knowledge about patients’ priorities and needs regarding oral nutritional supplement therapy. The discrete choice experiment is an innovative method used to elicit patients’ preferences. Good practice guidelines emphasize that the development of attributes and levels is a fundamentally important process.

**Objective:**

To comprehensively describe the identification, refinement, and selection of attributes and levels for a discrete choice experiment.

**Methods:**

A mixed-methods approach, consisting of three consecutive steps: a literature review, in-depth interviews, and focus groups. First, the literature review allowed quick identification of attributes and levels. Then, 15 in-depth interviews were conducted to gather a rich description of the experience of patients taking oral nutritional supplements after gastrectomy and to verify and enrich the attributes and levels list. Finally, four focus group participants discussed the wording of the attributes and levels and reduced the number of attributes to manageable numbers through voting ranking methods.

**Results:**

Following the literature review and qualitative data collection, eight attributes were finally generated, each with two to three levels. The following attributes were included: 1) information provider; 2) health guidance approach; 3) adverse reactions; 4) flavor; 5) follow-up method; 6) follow-up frequency; 7) psychological support; 8) cost. These attributes covered the important attributes of nutritional preparations and health guidance included in ONS therapy that were relevant to patients.

**Conclusions:**

This study’s mixed-methods approach has been found highly suitable to identify, refine and select attributes and levels for a discrete choice experiment. The three methods have pros and cons, and they complement each other, especially the analysis of qualitative data led to a deeper and broader understanding of attributes and levels.

## Introduction

Gastric cancer is a common malignant tumor of the digestive tract, the incidence ranks fifth in the world and the fourth most common cause of death from malignant tumors worldwide, especially in East Asia [1]. Currently, radical gastrectomy is still the main method of gastric cancer treatment [2]. However, resection of the stomach will reduce reservoir function and bring problems from the reconstruction of the structure of the digestive tract [3]. Studies have shown that 19.0–68.8% of patients after gastrectomy suffer from malnutrition [4]. The European Society for Clinical Nutrition and Metabolism (ESPEN) recommended that oral nutritional supplements (ONS) be given priority in nutritional therapy for patients suffering from malnutrition [5], a higher adherence to ONS can improve the nutritional status and immune function [6], reduce the incidence of complications [7], thus shortening the length of hospital stay [8]. However, the adherence of patients with ONS after gastric cancer surgery was only 26.2%-58.0% [9–11].

So far, more and more studies have attempted to provide a series of ONS with flavors, texture, and compositions according to patients’ preferences to improve adherence [12,13]. However, the patient’s adherence to the ONS therapy is affected by many factors, including types of therapeutic regimens, nutritional monitoring and guidance, and disease progression [14–16]. Given the needs of ONS to improve nutritional status and the diversity of adherence factors, a better understanding of patients’ preferences for treatment characteristics may help clinical staff to develop intervention strategies to improve adherence. Moreover, health professionals are increasingly encouraged to involve patients in treatment decisions [17]. However, there is little knowledge about patients’ priorities and needs regarding ONS therapy, and practical concerns include the extra time needed and the difficulties in eliciting patient preferences, which poses challenges for clinical staff.

The discrete choice experiment (DCE) is one innovative approach to overcoming this limitation, which the aim is to elicit patient preferences for healthcare interventions or products [18]. In recent years, several studies have used a DCE to elicit and compare preferences in terms of the attributes of home enteral nutrition among patients and physicians [19,20]. However, available measures were designed for tube feeding therapy for patients with chronic diseases other than cancer. In addition, these DCEs neglected the impact of health guidance and follow-up strategies other than nutritional products on patient adherence. Studies have shown that providing detailed information on the nutritional needs of patients by medical staff can increase the compliance of hospitalized patients by 41% to 67% [21]. A systematic review showed that ONS compliance was significantly higher in patients who received more follow-up and encouragement from healthcare professionals than in survey studies [22]. Therefore, a novel DCE is needed to investigate the preferences for ONS therapy among postoperative patients with gastric cancer, and to provide distinctive references for the development of ONS management strategies in the future.

Good practice guidelines emphasize that the development of attributes and levels is a fundamentally important process [23,24]. However, most DCE studies only described methods used to identify attributes and levels (e.g., literature reviews, interviews, and focus groups), and lacked detailed information about a systematic process to identify attributes and levels that were most salient and important for health care treatment decisions [18]. A comprehensive description of this process ensures transparency, thus allowing researchers to judge the quality and generalizability of a DCE [25]. Therefore, this study aimed to comprehensively describe the identification, refinement, and selection of attributes and levels for a DCE on preferences for ONS therapy among postoperative patients with gastric cancer, and to prepare for the design and implementation of the eventual discrete choice questionnaire.

## Methods

### Study design

The study was a single-center, exploratory study performed in real clinical practice in China. In this study, a mixed-method approach was used to develop attributes and levels. It consisted of three consecutive steps: 1) a literature review; 2) in-depth interviews, and 3) focus groups.

### Step 1: A literature review

Because the purpose of our review was to identify and map the research on attributes and levels, we decided to conduct a systematic scoping review of the literature [26]. The following steps were undertaken: (i) identifying the research question; (ii) identifying relevant studies; (iii) screening and selection of studies; (iv) charting the data; and (v) collating, summarizing, and reporting the results [27].

### Identifying the research question

The following research question was identified: what attributes and levels of ONS preference in patients after gastrectomy can be identified from the literature?

#### Identifying relevant studies

Databases including Embase, PubMed, Web of science, Chinese National Knowledge Infrastructure (CNKI), and Wanfang database were searched from the earliest available time up to October 2020. Key terms included “neoplasm”, “oral nutritional supplement”, “patient adherence” and “patient preference”. The search language was limited to English and Chinese. There were no restrictions on study designs. The detailed search strategy was shown in **(see S1 Table).**

#### Screening and selection of studies

After removing duplicates, two authors independently screened titles and abstracts of articles retrieved from the search strategy, and then assessed the eligibility of relevant full-text articles for inclusion in the review. The inclusion criteria were: 1) study on patients after gastrectomy aged 18 years or older; 2) study on adherence to ONS, preferences for ONS, or experiences with ONS. Those that did not meet the above criteria would be excluded. Disagreements were resolved through consensus among the two authors, with a third author as arbiter where required.

#### Charting the data and collating, summarizing, and reporting the results

After reading the full article of each study included in the scoping review, two authors charted the data. Key information extracted included: author(s) (year of publication, location), objective(s), study design, surgical procedure, sample size, and attributes (levels). Finally, based on the data chart, the researchers jointly compiled the initial list of attributes and levels.

### Step 2: In-depth interviews

#### Patient recruitment

To gather a rich description of the experience of patients taking ONS after gastric cancer surgery, patients were recruited in the gastrointestinal surgery and oncology department of a tertiary hospital in Changchun from October 2020 to December 2020. A purposeful sampling method was used to obtain the greatest difference in the experience of patients. Eligibility criteria were: 1) Patients with stage I, II, or III gastric cancer were diagnosed by pathology before surgery; 2) had distal or total gastrectomy; 3) Age≥18 years old; 4) currently receiving ONS or having received it during the previous year. If the patient had other malignant tumors, or had impaired consciousness and could not communicate normally, they would be excluded. All of the participants gave their written or verbal informed consent.

### Data collection

Due to the COVID-19 pandemic, the research team conducted interviews in two ways. The face-to-face interview took place in patients after gastrectomy who were re-hospitalized and needed adjuvant treatment. These patients underwent nucleic acid testing before admission, and met the COVID-19 epidemic prevention standards of China. Those who were recovering at home after gastrectomy were interviewed by telephone. Hospitalized patients were interviewed in a private room in the hospital. The interviewer received professional training before conducting the interview. To alleviate patients’ concerns about safety, the research team adopted standard prevention and control measures, such as wearing medical surgical masks, keeping physical distance between interviewers and participants, and disinfecting the room immediately after the interview. We formulated a semi-structured interview outline based on the initial attributes and levels obtained from the literature review and focused the discussion on topics related to personal ONS treatment experience. Topics included: 1) feelings and views of patients taking ONS; 2) related factors that affect patients’ adherence to ONS; 3) sources of help to promote patients’ adherence to ONS; 4) acceptability and availability of health services, including health guidance and out-of-hospital follow-up. The final interview guidelines were refined through pilot interviews **(see S2 Table).** Before the start of the interview, each patient completed a brief questionnaire on socio-demographic and clinical characteristics. All interviews were recorded with permission and transcribed verbatim. Saturation was achieved when no new attribute was identified.

#### Data analysis

Data collection and analysis proceeded concurrently, and the thematic analysis method was used to analyze the transcript [18]. The codes and themes were pre-selected based on the list of attributes and levels. Revision of the pre-selected codes and themes and the addition of new themes through the inductive method. Nvivo version 12.0 was used to manage the data [28].

### Step 3: Focus groups

Focus group participants provided feedback through short activities to verify the attributes from the in-depth interviews. An experienced independent host and an assistant host conducted the focus groups. The focus group was conducted in a private space in the hospital. We used a purposeful sampling method to recruit patients who were hospitalized in the oncology department in December 2020 and divided them into four focus groups according to the time of admission, each of which consisted of 4–5 participants. The selection criteria for participants and the COVID-19 prevention and control criteria were the same as in-depth interviews.

The focus group activities were divided into three parts. First, for each listed attribute and level, participants indicated the relevance to their experience, and individually supplemented attributes and levels that were important to them in making choices regarding ONS treatment. Second, a ranking exercise was performed to scale down the number of attributes to a number manageable within a DCE. The newly identified attributes also were ranked. These attributes were awarded points: from 3 points for the most important attribute to 1 point for the least important attribute. Per attribute, the mean importance score was then calculated by dividing the total awarded points per attribute by the total number of participants in all focus groups [29]. Participants were asked to individually rank the attributes by relevance, and the mean scores showed the group aggregate rank [30]. Based on the mean importance score, we made the ranking of attributes from most (highest mean) to least (lowest mean) important. Too many attributes increase the complexity of the task for participants which may increase the chance of inconsistent responses across choice tasks or participants not considering all the attributes when making a decision [31]. Thus, similar to the previous discrete choice experiment, we plan to include the top eight most important attributes in our follow-up study [30]. Third, a group discussion on the patients’ rankings and the wording of the attributes and levels were held. Researchers and focus group participants assess the consistency of the meaning and interpretation of the attributes. Group discussion continued until all attributes and levels were fully and clearly described.

The final list of attributes and levels was evaluated by an expert panel. The expert team consists of two gastric oncologists, two dietitians, two nutrition specialist nurses, and two researchers. The expert panel discussed and determined the range of the levels, and took into account their clinical plausibility.

### Ethics approval and consent to participate

The study was approved by the Ethics Committee of the Nursing School of the University of Ji Lin, China (No.2020082803) and completed registration in the Chinese clinical trial registry (registration number; ChiCTR2000041047). Informed consent was obtained from participants at the start of the survey. Written consent was provided by those participants taking part in interviews conducted face-to-face; verbal consent was provided and documented by the interviewer in telephone interviews where written consent could not be obtained. This consent procedure was approved by the ethics committee. Due to the COVID-19 pandemic, informed consent contains detailed prevention and control measures for face-to-face interviews, and patients have the right to refuse to participate in the survey due to risk considerations.

## Results

### Step 1: A literature review

**Fig 1:**
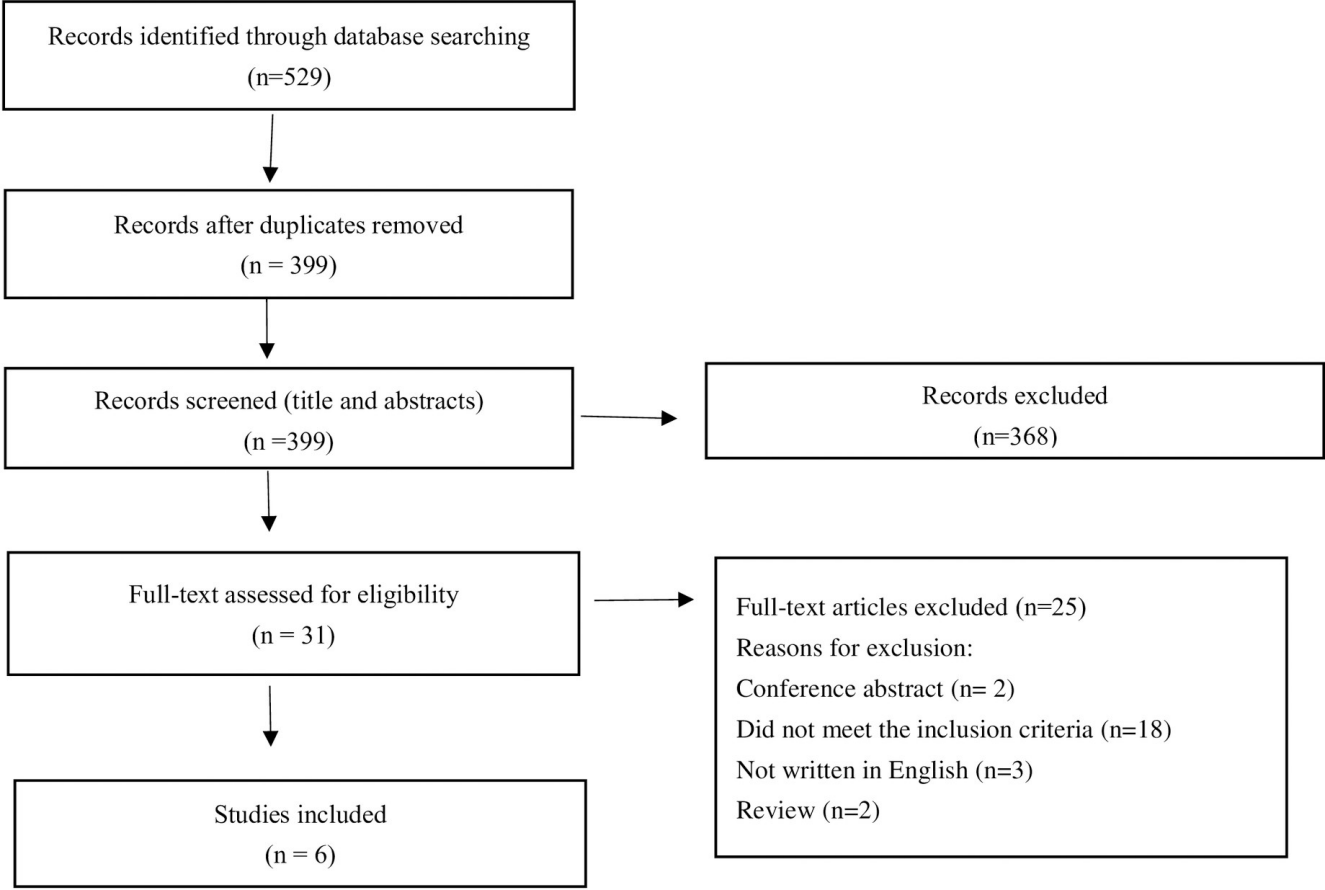
Flowchart selection process.

The search generated 529 articles (PubMed: n = 86; Embase: n = 131; Web of science: n = 237, CNKI: n = 55, Wanfang: n = 20). 130 duplicates were removed, yielding 399 records for eligibility screening. After screening on title and abstract led to the exclusion of 368 articles; 31 articles were read in full and assessed for eligibility, and an additional 25 were excluded. Finally, six studies met our inclusion criteria [15,32–36]. **Fig 1** shows the flowchart of the selection process. Data about the study features are charted in **Table 1.** According to the data graph, our research team evaluated the attributes and levels list obtained after generating the literature review and obtained 12 attributes. Since the literature did not reflect the specific value of the attribute of cost in detail, we could not analyze the level of this attribute. Although most articles mentioned that adverse reactions were the most common reason for patients to stop taking ONS, such as bloating and diarrhea, early satiety, and nausea, we could not assess the patient’s acceptance of the degree of risk from the literature [10,15]. Therefore, in addition to the attribute “cost” and the attribute “adverse reactions”, each of the remaining attributes had two to four levels. **Table 2** shows the detailed information on the initial attributes and levels.

**Table 1:** Study characteristics.

Study	Objective	Location	Study design	Sample size(n)	Pathological stage	Surgical procedure	Adjuvant treatment	Attributes(levels)
Copland et al. [35] (2007)	To evaluate the effects of individualized oral nutritional support a long time after total gastrectomy	Sweden	Quasi-experiment study	15	NA	Total gastrectomy	NA	• Dietitian • Monthly at the regular follow up • Contact patients over the telephone • A energy powder supplement or liquid supplement or a hot soup • Intake
Zhao et al. [32] (2018)	Acquired the understanding and feeling of the patients with gastrointestinal tumors after surgery and receiving oral nutrition powder after adjuvant chemotherapy	China	Qualitative study	14	Ⅰ II III	Total gastrectomy; Distal gastrectomy	Chemotherapy	• The aroma and flavors are too sweet • Supervision and guidance • The reason for taking ONS is unknown • Cost • Adverse gastrointestinal reactions • Ways to pay attention to health information • Self-awareness and attitude
Liljeberg et al. [34] (2019)	To assess adherence to ONS among hospital outpatients and to assess patient characteristics, experiences of ONS, and the characteristics of ONS prescriptions in clinical practice.	Sweden	Cross-sectional study	51	NA	NA	NA	• Flavors • The duration of ONS • Prescribing dietitian • Prescribed amount • Support from others • Regarded ONS as food rather than medicine • Timing of ONS consumption • Nausea, satiety, fullness, texture, swallowing difficulties, stomach pain • Convenience of taking ONS • Monitored by healthcare professionals
Ma et al. [33] (2019)	To describe the nutritional status and the application of home enteral nutrition support in patients with gastric cancer four weeks after discharge, and to identify the problems in home enteral nutrition support during the period.	China	Non-experimental observational study	124	Ⅰ II III Ⅳ	Total gastrectomy; Distal gastrectomy	Chemotherapy	• The nutrient solution tastes bad • Gastrointestinal intolerance • Cost • Regular follow-up to answer questions or supervise • Single taste • Patient education
Chu et al. [36] (2020)	To explore the effect of feedback teaching in the health education of ONS for discharged patients after gastrointestinal tumor surgery	China	Quasi-experiment study	26	II III	Total gastrectomy; Distal gastrectomy	Chemotherapy	• Feedback teaching intervention team (nurse specialist, attending physician, dietitian) • Telephone follow-up
Lidoriki et al. [15] (2020)	To explore postoperative compliance with ONS and define barriers to consumption in patients with esophageal, gastroesophageal junction, and gastric cancer.	Greece	Cross-sectional study	78	Ⅰ II III Ⅳ	Total gastrectomy; Distal gastrectomy	Chemotherapy	• Bloating • Early satiety • Flavor or texture dislike • Diarrhea • Patient education and support • Subjectively not needing the supplements • Individual follow-up time • Strategic timing of oral nutrition support • The prescribed amount of ONS • Postoperative monitoring

*Note*. ONS: Oral nutritional supplement; NA: No data or not described.

**Table 2:** Initial attributes and levels obtained from the literature review.

Serial number	Attributes	Levels
1	Flavor	1. Single taste of ONS provided; 2. A variety of ONS flavors available; 3. The provided ONS tastes bad; 4. The provided ONS tastes good
2	Formulation type	1. Powder 2. Liquid
3	The duration of ONS	1. One month 2. Three months 3. Six months
4	Timing to supplement ONS	1. Before meal 2. Between meals 3. After meal
5	Cost	----
6	Adverse reactions	----
7	Information provider	1. Dietitian 2. Attending physician 3. Nurse specialist
8	Follow-up frequency	1. Once a week 2. Once a month
9	Follow-up method	1. Via telephone 2. Via outpatient
10	Self-perception	1. Patients know the reason and importance of taking ONS 2. Patients are not clear about the reason and importance of taking ONS
11	Convenience	1. I feel that ONS is very convenient to take 2. I feel that ONS is very troublesome to take
12	Social support	1. Healthcare professionals encourage me to stick to ONS 2. Other patients experiencing similar illnesses encourage me to stick to ONS 3. The caregiver who lives with the patient encouraged me to stick to ONS

*Note*. ONS: Oral nutritional supplement.

## Step 2: In-depth interviews

### Description of the sample

The 15 patients participated in one-to-one in-depth interviews, of which eight patients were interviewed face-to-face, and seven patients were interviewed by telephone. The interviews lasted between 30 and 50 minutes. **Table 3** shows the socio-demographic and clinical characteristics of the patients.

**Table 3:** Patients’ sociodemographic and clinical characteristics.

Variable	In-depth interviews (n = 15)	Focus groups (n = 19)				
		Group 1 (n = 4)	Group 2 (n = 5)	Group 3 (n = 5)	Group 4 (n = 5)	Total (n = 19)
**Gender, n (%)**						
Male	7(46.7)	1(25.0)	2(40.0)	2(40.0)	2(40.0)	7(36.4)
Female	8(53.3)	3(75.0)	3(60.0)	3(60.0)	3(60.0)	12(63.2)
**Age (years), mean (SD or range)**	55.9±12.4	53(42–66)	60(52–64)	52(38–70)	55(34–75)	55(34–75)
**Education level, n (%)**						
Primary school or below	4(26.7)	0	1(20.0)	0	1(20.0)	2(10.5)
Junior high school	5(33.3)	1(25.0)	3(60.0)	3(60.0)	1(20.0)	8(42.1)
High school	1(6.7)	0	1(20.0)	0	0	1(5.3)
College or university	5(33.3)	3(75.0)	0	2(40.0)	3(60.0)	8(42.1)
**Caregivers, n (%)**						
The patient himself	1(6.7)	0	1(20.0)	1(20.0)	0	2(10.5)
Children or spouse	8(53.3)	4(100.0)	3(60.0)	4(80.0)	5(100.0)	16(84.2)
Both children and spouses	6(40.0)	0	1(20.0)	0	0	1(5.3)
**Household per capita monthly income, ¥, n (%)**						
< 1000	3(20.0)	1(25.0)	0	1(20.0)	1(20.0)	3(15.8)
1001–3000	4(26.7)	1(25.0)	2(40.0)	0	1(20.0)	4(21.1)
3001–5000	3(20.0)	1(25.0)	3(60.0)	0	1(20.0)	5(26.3)
> 5000	5(33.3)	1(25.0)	0	4(80.0)	2(40.0)	7(36.8)
**Pathological stage, n (%)**						
I	3(20.0)	0	0	0	0	0
II	3(20.0)	2(50.0)	1(20.0)	2(40.0)	2(40.0)	7(36.8)
III	9(60.0)	2(50.0)	4(80.0)	3(60.0)	3(60.0)	12(63.2)
**The surgical procedure, n (%)**						
Distal gastrectomy	11(73.3)	3(75.0)	3(60.0)	4(80.0)	2(40.0)	12(63.2)
Total gastrectomy	4(26.7)	1(25.0)	2(40.0)	1(20.0)	3(60.0)	7(36.8)
**The texture types of ONS, n (%)**						
Liquid	3(20.0)	0	2(40.0)	2(40.0)	1(20.0)	5(15.8)
Powder	12(80.0)	4(100)	3(60.0)	3(60.0)	4(80.0)	14(84.2)
**Duration of taking ONS, n (%)**						
< 1month	5(33.3)	1(25.0)	0	3(60.0)	1(20.0)	5(26.3)
1-3months	2(13.3)	0	1(20.0)	0	1(20.0)	2(10.5)
3-6months	4(26.7)	2(50.0)	3(60.0)	0	1(20.0)	6(31.6)
> 6months	4(26.7)	1(25.0)	1(20.0)	2(40.0)	2(40.0)	6(31.6)

*Note*. SD: Standard deviation; ONS: Oral nutritional supplement.

### Attributes and levels identified from in-depth interviews

After in-depth interviews, three new attributes were discovered: “purchase route”, “ways to obtain information”, and “health guidance approach”. Participants said that they would consider whether it was easy to buy products when they were undergoing ONS therapy, which was also an important factor in deciding that they continued to take ONS for a long time. On the other hand, due to the shortened hospital stay, patients hoped to learn about oral nutrition therapy through multiple channels, such as expert lectures, diet manuals, etc., and more hoped for targeted one-to-one guidance, although at present, they could get general information from various bulletin boards in hospital wards.

In addition, we modified the attribute “the duration of ONS”. In the interview, the patient stated that although the attending physician recommended taking at least three months to meet the physical needs during the hospitalization, the duration of follow-up by medical staff may affect the length of time they ultimately take ONS. Therefore, our research team believes that the attribute of “duration of follow-up” is more relevant to patients.

Based on the content of the interview, we also revised some levels of attributes. Participants said that they were concerned about the degree of adverse reactions during ONS therapy, and frequent adverse reactions were the main reason they stopped taking ONS. Therefore, we set the level of the attribute of "adverse reactions" as the frequency of adverse reactions, that is, “almost none, occasionally, often”. Similarly, for the attribute of “social support”, the interviews found that there were differences in the degree of social support among participants during ONS therapy, while the people who supported them were similar. Therefore, we revised the level of this attribute from the original “source of social support” to “the degree of social support.”

Besides, we also added some attribute levels. For example, the attribute of the “follow-up method” adds the level of “via WeChat”, which is currently the most popular smartphone application in China for messages. **Table 4** presents exemplary quotes for each of the 14 attributes identified from the analysis of the in-depth interviews. Except for the attribute “timing to supplement ONS” in the literature review, other attributes have been verified by the interviews. So, the focus group discussed 15 attributes.

**Table 4:** Included attributes and levels from in-depth interviews.

Attributes	Levels	Quote excerpted from transcribed interviews
Flavor	1. Single taste of ONS provided 2. A variety of ONS flavors is available 3. The provided ONS tastes bad 4. The provided ONS tastes good	*“The taste*, *some patients said it tasted like milk powder*, *and some said it tasted like rust*. *It can’t be said*, *the taste is not particularly good”* *“If there were multiple flavors*, *such as ice cream flavor*, *I like sweet ones*, *and I would continue to drink them*.*”* *“I feel greasy after eating for a long time*, *and the taste needs to be improved*. *It has no sweet taste*. *The more you drink it*, *the worse it tastes*. *Do you want to say that the taste is acceptable*? *You can only bite the bullet and drink it*.*”*
Formulation type	1. Powder 2. Liquid	*“When I was discharged from the hospital*, *it was said that there was a liquid ONS*. *I wondered if the bottle would be difficult to store once I opened it*. *I chose powder instead”* *“I was thinking about taking powders for nutritional supplements*. *The bottled nutritional supplements are not easy to store after opening*.*”* *“I’m drinking a liquid nutrient because the powder nutrient is not easy to count calories”*
Adverse reactions	1. When I took ONS, there was almost no gastrointestinal discomfort 2. I occasionally feel gastrointestinal discomfort when I take ONS 3. I often experience gastrointestinal discomfort when I take ONS	*“After the operation*, *I tried many kinds of oral nutrition*, *both powder*, *and liquid*, *but I felt nauseous every time I ate it*, *so I didn’t dare to continue drinking it*.*”* *“During the period of taking it*, *some patients had diarrhea*, *which I have never encountered*, *and it’s okay if I drink it quickly and slowly*.*”* *“If I had diarrhea as soon as I drank it*, *I did not want to drink it*, *but occasionally several times uncomfortable*, *I could accept*.*”* *“I drank it within a few days after the operation*, *but after I drank it*, *I couldn’t tolerate it*, *nausea and vomiting*, *I didn’t dare to drink it at all*, *and I dare not drink it now*.*”* *“I was a little nauseous at the beginning*, *but later adjusted the method to drink slowly and sip*, *and then I feel comfortable*. *After a long time*, *I gradually get used to it*.*”*
Cost	----	*“If the price is lower*, *I can continue to take some nutritional powder*. *After all*, *I am sick*, *and I have to plan to spend money on all aspects*.*”* *“I have pension and employee medical insurance*, *which can help me to reimburse part of the cost of ONS therapy*. *In addition*, *my child has a well-paid job and occasionally sends me some other kinds of ONS*, *so I did not bear great financial pressure during the ONS therapy”* *“After the operation*, *my body was in a weak state*. *I urgently want to get nutrition guidance from my attending physician on how to better recover my physical activity*. *I care more about my body than the cost of nutrition therapy*.*”*
Purchase route	1. Hospital 2. Nearby pharmacy 3. Online Shopping	*“Although I can buy it online and on JD*.*com*, *I need to be hospitalized every month*. *I think it’s safer to be prescribed by the physician in the hospital*.*”* *“It seemed that my child bought online was cheaper than at the hospital*, *and I can still accept it in terms of price*.*”* *“It is more convenient for me to prescribe nutrients in the hospital*. *Medical insurance can help me reimburse part of it*. *For reimbursement*, *I don’t buy them in pharmacies or online*.*”*
Convenience	1. I feel that ONS is very convenient to take 2. I feel that ONS is very troublesome to take	*“Sometimes I may find it inconvenient to eat*, *and I have to stir and mix it into a liquid*, *so I don’t want to do it*.*”* *“The nutrient of the powder is not good*. *two scoops at a time are a bit thick*, *and 1 scoop at a time is a bit weak*. *It is not easy to calculate energy*. *You have to stir and mix with warm water*. *It is not very convenient to carry out*.*”* *“I think this nutrient is very convenient to use*, *is it the same as drinking milk powder*?*”*
Information provider	1. Dietitian 2. Attending physician 3. Nurse specialist	*“The physician participated in my operation*. *If he wants me to drink some nutrients to supplement my nutrition*, *I have to drink it obediently as a medicine*.*”* *“If I don’t have the guidance of a dietitian at home*, *I don’t know where to get this knowledge*. *The physician mainly cares about our diseases*, *and he doesn’t know nutrition well*.*”* *“Later*, *during the follow-up visit outside the hospital*, *the nurse told me to drink slowly*, *slow down*, *and sip*. *I followed her method and there would be no discomfort*.*”*
Ways to obtain information	1. Expert live lecture 2. Dietary instruction manual 3. Network platform (such as WeChat discussion group)	*“Now I want to know how I can eat more comfortably*, *be healthier*, *and recover faster*. *If there are rehabilitation lectures by experts in this area*, *I would be willing to listen to them*.*”* *“The nutrition WeChat group helped me avoid many detours*. *The instructional video was posted in the group*, *which was more comprehensive*.*”* *“In my idealized state*, *it’s better to have some books in electronic version*, *which are relatively easy to understand*.*”*
Health guidance approach	1. One-to-one 2. Group education	*“One-on-one consultation is best*. *After all*, *each person’s body constitution is different*, *but it seems unrealistic*. *After all*, *human resources are limited and the number of our patients is relatively large*.*”*
Follow-up frequency	1. Once a week 2. Once every 2 weeks 3. Once a month	*“Hope to provide regular help*, *preferably once a month*, *or once every 2 months*. *During the follow-up*, *I feel that this has been cared for by the medical staff”* *“I hope that the hospital will communicate directly with patients at least once a month*.*……*.*”* *“For patients who don’t know how to popularize science*, *you (the hospital) will return to visit*. *For example*, *after two weeks*, *follow-up in the first two weeks is unnecessary*, *because the dietary guidance provided by discharge is sufficient to meet the needs*.*”*
Follow-up method	1. Via telephone 2. Via outpatient 3. Via WeChat	*“I am afraid that it will affect your work*. *I don’t know a lot of things if I don’t call*. *I can directly consult if I have any ideas*. *After all*, *I am over 50 years old*, *my eyes are stretched*, *I can’t see clearly*, *and high-tech things such as the Internet can be difficult for me*.*”* *“I like outpatient follow-up*. *This face-to-face way is more beneficial to your work*. *It can see the mental outlook of the whole person for the first time*, *and it can also conduct various inspections and give more objective suggestions*.*”* *“If our hospital has a WeChat official account*, *it would be better to push related videos*. *This kind of establishment of a WeChat group can be consulted online*, *even if a certain fee is charged*, *I am quite willing*.*”*
Duration of follow-up	1. One month 2. Three months 3. Six months	*“Communicate with me once a month or two months*. *I think it’s good to communicate four or five times*.*”* *“I consulted two or three times when I first started a month*. *Two months later*, *the teaching video was posted in the group*, *which was more comprehensive*. *There was no problem if I followed the instructions and did not consult*.*”*
Self-perception	1. Patients know the reason and importance of taking ONS 2. Patients are not clear about the reason and importance of taking ONS	*“At first*, *I was resistant to using nutrients*. *Later*, *my wife and daughter carefully introduced me to the composition of nutrients and explained the difference between nutrients and millet soup*. *I eliminated the resistance from my heart and accepted the nutrients emotionally*.*”* *“I stopped taking the nutritional powder for half a year*. *I mainly felt that there was no nutrition and the weight was not maintained*.*”*
Social support	1. Almost no one discusses ONS with me 2. Remind me occasionally to conduct ONS 3. Always encourage me to take oral nutritional supplements	“My child wants me to continue to drink this, saying that when I have a bad appetite, drinking this is better for me, and I don’t want to burden them.” “I tried to stop it several times, but it was the dietitian wan who reminded and persuaded me in time, so I insisted on using it for more than three months.”

*Note*. ONS: Oral nutritional supplement.

### Step 3: Focus groups

#### Description of the sample

Four focus groups including 19 participants discussed attributes and levels related to their experiences, among them, five patients with gastric cancer had participated in in-depth interviews before. The duration of the focus group was about 60 minutes. **Table 3** shows the socio-demographic and clinical characteristics of the patients.

### Attributes and levels identified from focus groups

Participants in the focus group did not discover new attributes and levels. After the first focus group, participants suggested combining the attribute “convenience” and the attribute “formulation type”. They said that the attribute of “convenience” actually expressed their preference for different types of ONS. In other words, when patients expressed their preference for powder packaging, the convenience of powder packaging was more attractive to them. In addition, the participants proposed that the attribute “social support” contained more extensive content, and they expected more emotional support, so it was recommended to change this attribute to “psychological support”. Besides, it is recommended that the level of “psychological support” attribute be set to whether or this support is provided.

The second and third focus groups suggested removing the two attributes of “self-perception” and “ONS taking time”. Participants stated that when participating in ONS therapy, the medical staff would tell them the prescribed time to take ONS in advance, and could flexibly adjust it according to their eating habits. In addition, participants believed that before they chose ONS, their attending physician would explain in detail the reasons and importance of taking ONS, and professional health guidance would improve their self-perception. Therefore, the content reflected by the attribute of “self-perception” and the “ONS taking time” can be included in another attribute, namely “information provider”.

After the fourth group, the participants said that it was more appropriate to focus on the taste of the patient’s experience because the popularity of online shopping made it easy for them to try different types of ONS products instead of a single product in the hospital. Therefore, the level of the attribute “flavor” is reduced to two.

In the ranking task, participants voted on 12 attributes. The detailed information of the voting results was shown in **(see S3 Table)**. The top seven most important attributes include three parts: health guidance, follow-up strategy, psychological support and experience attributes closely related to ONS therapy. The attributes of “information provider” and “health guidance approach” attained the highest scores in rating. The attribute “cost” does not score high in the voting rankings, and only ranks 11th. However, the expert panel stated that the results of the qualitative analysis showed that the cost attribute was relevant from the perspective of patients after gastrectomy. In the in-depth interview, there were two voices on cost attribute: patients with lower family economic income were under certain economic pressure when taking ONS for a long time, compared with patients with higher income; gastric cancer patients were in a weak state in the early stage after surgery, and their attention was focused on postoperative rehabilitation rather than the cost of ONS. On the other hand, by adding continuous variables to the DCE, participants’ willingness to pay can be obtained [37]. Thus, the results of the ranking exercise and qualitative analysis led to the following attributes: 1) information provider; 2) health guidance approach; 3) adverse reactions; 4) flavor; 5) follow-up method; 6) follow-up frequency; 7) psychological support; and 8) cost.

Finally, the expert panel identified and refined the levels of eight attributes. The level “once a month” of the attribute “follow-up frequency” was modified to" once every 4 weeks”. The level of the attribute “adverse reactions” has been refined to “almost none”, “occasionally" and “often”. The original description was too long, easy to cause visual fatigue participants. The level of cost was based on the current price of ONS preparations commonly used in hospitals and the market, and the weekly cost of ONS is calculated according to the consumption of 400–600 kcal per day as prescribed by the attending physician. According to the type of ONS taken by patients, it varies from 200 RMB to 600 RMB, with an average of about 400 RMB. The final attributes and their levels are presented in **Table 5.**

**Table 5:** List of final attributes and levels.

Attributes	Levels	Description of the attributes
Information provider	Dietitian Attending physician Nurse specialist	Professionals who provide health guidance on the effects of oral nutritional supplements, how to take them, coping strategies for adverse reactions, nutritional monitoring, and follow-up when patients need to take oral nutritional supplements when they suffer from malnutrition.
Health guidance approach	One-to-one Group education	One-to-one refers to the targeted individual guidance of individual patient when professionals provide health guidance; Group education refers to the targeted and focused education of patients with similar needs when professionals provide health guidance.
Adverse reactions	Almost none Occasionally Often	The degree of gastrointestinal intolerance such as fullness, bloating, nausea, vomiting, and abdominal pain when taking oral nutritional supplements.
Flavor	Good taste Bad taste	The taste experience when taking oral nutritional supplements may be related to the formula, appearance, smell, texture, etc. of the food itself.
Follow-up method	Via outpatient Via telephone Via WeChat	Tools used by professionals to follow up with patients during the period of taking oral nutritional supplements.
Follow-up frequency	Once a week Once every 2 weeks Once every 4 weeks	The number of follow-up visits per unit time by professionals during the period of taking oral nutritional supplements;
Psychological support	Yes No	During the period of taking oral nutritional supplements, whether professionals provide additional guidance such as emotional support and psychological counseling.
Cost (RMB/week)	200 400 600	The average weekly cost of purchasing nutritional supplements for patients taking oral nutritional supplements.

## Discussion

Given the poor adherence to ONS therapy among patients after gastrectomy, DCE was used to explore the crucial factors that could affect patients’ preferences, allowing either possible product improvements or therapeutic focused adjustments. This study was the first attempt to develop attributes and attribute levels on preferences for ONS therapy among patients after gastrectomy. We tried to comprehensively describe the development process of attributes and levels according to the principles used in DCE studies.

Previous studies address therapy preferences globally, such as dosing schedules and duration of effect, or specifically, the preference characteristics of these pharmaceutical services are usually not “modifiable” [38,39]. The preference for ONS in patients after gastrectomy is related to more general attributes, such as frequency of follow-up and psychological support. To our knowledge, there was currently no standardized ONS management strategies, which may be related to limited medical resources and cost-effectiveness [40]. To a certain extent, our study provides the research ideas of patients’ preference for nutrition therapy and compliance, and identifies key attributes that affect ONS compliance of gastric cancer patients after surgery from the perspective of patients.

In this study, the attributes and levels were developed using a mixed-method approach. This study’s mixed-methods approach has been found highly suitable to identify, refine and select attributes and levels for a DCE. The literature review allowed a quick identification of attributes and levels. In this study, we searched not only literature related to the preference category, but also literature related to ONS compliance. This approach provides a new idea for formulating the search strategy of DCE. However, identifying attributes and their levels exclusively on the basis of a literature review may lead to the non-inclusion of some important attributes [25]. The in-depth interview of the second step made it possible for the researchers to gain a deeper insight into the relevance of the attributes and levels from the view of patients. Three new attributes were identified in the in-depth interview, which supplemented the list and also verified the effectiveness of the previous search strategy. During the focus groups, the patients discussed the wording of the attributes and levels. This resulted in the inclusion of attributes and levels that are understandable to the DCE’s target group. This highlights the importance of conducting qualitative work in the development of DCEs.

Exploratory work has a large number of attributes and levels. Due to the limitation of sample size and the potential cognitive burden of respondents, it is usually impractical to include all possible attributes and levels, and the statistical ability of DCE detection results will be reduced, usually reducing the number of attributes to manageable in the experiment [31]. Although there is no fixed threshold number, a previous systematic review found that most research attributes are between 2–12 [41]. Hiligsmann et al. suggested that using a simple ranking exercise may be sufficient for this purpose [42]. However, they stated that qualitative reasoning would still be required to guarantee relevant attributes and levels. This statement has been confirmed in this study, among the eight attributes finally selected, the attribute “cost” ranks not high. However, the expert panel still included the cost attribute according to the qualitative analysis results. A longitudinal study found that the patient’s financial ability during ONS therapy was independent factors that affected patient compliance in China [11]. As China’s medical insurance system limited the scope of payment for nutritional supplements or ONS, patients still bore high costs outside of medical insurance, and long-term use will increase the economic burden [43]. In the future, the attribute “cost” will provide objective data for medical policymakers to expand the reimbursement ratio of medical insurance.

Although we have reduced the number of DCE attributes as much as possible to reduce the cognitive burden of patients, eight aspects should be considered in each choice situation, and the requirements for patients to make choices are very high. Mangham et al. suggested that pictures were useful to explain attributes in a low- or middle- income country context where literacy cannot be assumed [44]. Considering that our sample was mostly middle-aged and elderly, visual elements may still help by reducing potential boredom and helping respondents engage.

This study has several strengths. First, shows how to rigorously and systematically conduct and report the process of deriving attributes and levels. This improves transparency and makes it reproducible. Secondly, our study used a mixed-methods approach to develop attributes and levels. The three methods have pros and cons, and they complement each other, especially the analysis of qualitative data led to a deeper and broader understanding of attributes and levels.

On the contrary, the study had some limitations. First, this study only included patients from the Department of Oncology and Gastrointestinal Surgery of a hospital in Northeast China, which may not be representative of other patients in China. It is possible that access experience and treatment availability could differ in other locations. In addition, the differences in education and income of patients in different regions were likely to affect patients’ preferences and priorities for ONS therapy. The education of the patients in the interview sample included in this study is mainly junior high school and university education. However, in a multicenter study on gastric cancer follow-up preference conducted by our team, the education of the included sample population is mainly senior high school education [45]. Similarly, compared with the more developed cities in southern China, the per capita household income of patients in Northeast China is lower and they may not like outpatient follow-up because they need to bear additional travel and accommodation costs [45]. In future studies, it is necessary to further confirm the information on the preference of Chinese patients after gastrectomy on ONS.

Second, although we use a purposive sampling approach to obtain a full range of views, the participants may not have disclosed all of their personal experiences and so some issues may have been missed. However, in the focus group discussion, the newly participating patients did not add new attributes and levels. Due to the impact of COVID-19, although we tried to recruit patients with different pathological stages, it is regrettable that only three patients with pathological stage Ⅰ participated in the in-depth interview without focus group. Four to six weeks after gastrectomy is the peak period of chemotherapy, as important adjuvant treatment in late pathological stage (e.g., Stage Ⅱ and III), which can affect the patient’s normal taste while suppressing tumor cells [11,46]. Compared with patients with early pathological stage, patients with late pathological stage may give the attribute “flavor” a higher ranking. In the next empirical study of DCE, it is necessary to explore subgroup analysis of patients at different pathological stages to explore the differences in the preferences of key attributes of ONS therapy, so as to provide personalized ONS management strategies.

## Conclusions

This study contributes to DCE literature by rigorously conducting and reporting the process of attribute development and level selection. Moreover, the suitability of a mixed-methods approach was highlighted. The effectiveness of DCE depends to a large extent on the ability of researchers to set relevant attributes and levels. Future research should pay more attention to a comprehensive description of this process. This is because the rigorous reporting of the attribute and level development process can improve the transparency of the design process, and help experts in related fields to judge the quality and versatility of DCE.

## Supporting information

S1 Table Search strategy. (DOCX)

S2 Table The final interview guidelines. (DOCX)

S3 Table Attributes voting results. (DOCX)
