# Supplementary material for: Preferences of oral nutritional supplement therapy among postoperative patients with gastric cancer: Attributes development for a discrete choice experiment
Source: PLoS One. 2022 Sep 29;17(9):e0275209. doi: 10.1371/journal.pone.0275209 (PMC9522277; doi:10.1371/journal.pone.0275209)
Supplement: S1 Table — (DOCX) [file pone.0275209.s001.docx]

**S1 Table** **Search strategy**

| **Literatur****e database** | **PubMed** | **Web of science** | **Embase** |
| --- | --- | --- | --- |
| **Search strategy** | #1 neoplasm OR cancer OR tumor  #2 “oral nutritional supplement*” OR “oral supplement*” OR “oral supplementation” OR “oral nutrition*” OR FSMP OR “sip feed*” OR “nutrition support” OR “nutrition intervention*” OR “nutritional intervention*” OR “nutritional support” OR “enteral nutrition” OR “nutrition therapy”  #3 "patient compliance"[Mesh] OR patient preference [Mesh]  #4 #1 and #2 and #3 | #1TOPIC:(neoplasm) OR TOPIC:(cancer) OR TOPIC: (tumor)  #2 TOPIC: (oral nutritional supplement*) OR TOPIC: (oral supplement*) OR TOPIC: (oral supplementation) OR TOPIC: (oral nutrition*) OR TOPIC: (FSMP) OR TOPIC: (sip feed*) OR TOPIC: (nutrition support) OR TOPIC: (nutrition intervention*) OR TOPIC: (nutritional intervention*) OR TOPIC: (nutritional support) OR TOPIC: (enteral nutrition) OR TOPIC: (nutrition therapy)  #3 TOPIC:( patient compliance) OR TOPIC:(patient preference)  #4 #1 and #2 and #3 | #1 ‘neoplasm’ ab,ti OR ‘cancer’ ab,ti OR ‘ tumor’ ab,ti  #2'nutrition therapy':ab,ti OR 'enteral nutrition':ab,ti OR 'nutritional support':ab,ti OR 'nutritional intervention*':ab,ti OR 'nutrition intervention*':ab,ti OR 'nutrition support':ab,ti OR 'sip feed*':ab,ti OR 'fsmp':ab,ti OR 'oral nutrition*':ab,ti OR 'oral supplementation':ab,ti OR 'oral supplement*':ab,ti OR 'oral nutritional supplement*':ab,ti  #3 ‘patient compliance’/exp OR ‘patient preference’ /exp  #4 #1 and #2 and #3 |
| **T****otal articles identified** | 87 | 244 | 136 |
