# Supplementary material for: Preferences of oral nutritional supplement therapy among postoperative patients with gastric cancer: Attributes development for a discrete choice experiment
Source: PLoS One. 2022 Sep 29;17(9):e0275209. doi: 10.1371/journal.pone.0275209 (PMC9522277; doi:10.1371/journal.pone.0275209)
Supplement: S2 Table — (DOCX) [file pone.0275209.s002.docx]

**S2 Table** **The final interview guidelines**

| Introduction | How is your recovery after surgery? What are the current diet and nutritional status? |
| --- | --- |
| Exploration | 1. Could you describe how you started taking oral nutritional supplements?  2. What is the reason for you to continue taking it as directed by your doctor? Or why did you stop taking it?  3. What do you think will help you keep taking oral nutritional supplements for a longer period of time?  4. How did you solve the problems you encountered while taking oral nutritional supplements?  5. What do you think of the current health guidance on oral nutritional supplements? Which aspects are most helpful for you to insist on taking ONS outside the hospital?  6. What other needs and suggestions do you have for the health guidance of oral nutritional supplements? |
| Ending | Anything else you’d like to add that we haven’t covered? |
