# Supplementary material for: Preferences of oral nutritional supplement therapy among postoperative patients with gastric cancer: Attributes development for a discrete choice experiment
Source: PLoS One. 2022 Sep 29;17(9):e0275209. doi: 10.1371/journal.pone.0275209 (PMC9522277; doi:10.1371/journal.pone.0275209)
Supplement: S3 Table — (DOCX) [file pone.0275209.s003.docx]

**S3 Table Attributes voting results**

| Attributes | Very important, n (%） | Generally important, n (%） | Unimportant, n (%） | Average score |
| --- | --- | --- | --- | --- |
| Information provider | 17(89.5) | 2(10.5) | 0 | 2.89 |
| Health guidance approach | 17(89.5) | 2(10.5) | 0 | 2.89 |
| Adverse reactions | 12(63.2) | 7(36.8) | 0 | 2.63 |
| Flavor | 11(57.9) | 8(42.1) | 0 | 2.58 |
| Follow-up method | 13(68.4) | 4(21.1) | 2(10.5) | 2.58 |
| Follow-up frequency | 12(63.2) | 5(26.3) | 2(10.5) | 2.52 |
| Psychological support | 11(57.9) | 7(36.8) | 1(5.3) | 2.52 |
| Ways to obtain information | 11(57.9) | 6(31.6) | 2(10.5) | 2.47 |
| Duration of follow-up | 11(57.9) | 6(31.6) | 2(10.5) | 2.47 |
| Formulation type | 9(47.3) | 7(36.8) | 3(15.8) | 2.32 |
| Cost | 8(42.1) | 8(42.1) | 3(15.8) | 2.26 |
| Purchase route | 7(36.8) | 9(47.3) | 3(15.8) | 2.21 |
